# Food the Best Physic

**Published:** 1855-11

**Authors:** 


### FOOD THE BEST PHYSIC.

An inseparable attendance on good health, is the regular daily action of the bowels; more than this, speedily induces debility; less, causes inaction, dulness, headaches, fevers, and death.

There is perhaps no one living whose bowels are not made free or costive by particular articles of food; the same article affects different persons variously. Each man must therefore observe for himself what articles constipate, and what loosen and act accordingly; a world of suffering and multitudes of lives would be saved every year by a proper attention to this simple suggestion, but not one man or woman in a thousand will give it that attention, hence the great mass of humanity perishes before its prime.

There are some articles of food which have various effects according to the parts used. The *May Apple*, or "Mandrake," is a nutritious fruit; its root is cathartic, its leaves a poison. The common house grape is a luscious product: the pulp is a delicious food, and in health should be the only part swallowed; the seeds loosen the bowels, while the skin constipates them. Two or three pounds of freshly picked, ripe grapes, may be eaten daily by a person in good health. The best time for eating them is immediately after breakfast and dinner.

The only safe, as well as the most rational practice of physic, is to make our food subserve medical uses. Knowing this, a doctor no more takes his own pills than an attorney goes to law, or a divine practises his own preaching

